# Microalgal bioactives as emerging nutritional regulators of apoptosis and neuroplasticity: targeting metabolic dysfunction in aging, neurodegenerative diseases, and cancer

**DOI:** 10.3389/fphar.2025.1643426

**Published:** 2025-11-12

**Authors:** Aili Wang, FenTao Bai, Jing Hua

**Affiliations:** 1 Department of Oncology, The Third Affiliated Hospital, Beijing University of Chinese Medicine, Beijing, China; 2 General Outpatient Clinic, The Third Affiliated Hospital, Beijing University of Chinese Medicine, Beijing, China

**Keywords:** microalgae, apoptosis, neurodegenerative diseases, cancer, molecular pathways

## Abstract

Microalgae are a rich source of multifunctional bioactive metabolites, such as carotenoids, polyunsaturated fatty acids, phycobiliproteins, and phenolics, that influence cellular metabolism, redox balance, and gene expression. Evidence from *in vitro*, *in vivo*, and limited clinical studies shows their dual capacity to support neuronal survival and plasticity while inducing apoptosis in cancer cells. In neurodegenerative diseases, these metabolites improve mitochondrial function, reduce neuroinflammation, enhance neurotrophic factor expression, and promote synaptic remodeling. In cancer, they trigger cell-cycle arrest, inhibit angiogenesis and metastasis, and activate both intrinsic and extrinsic apoptotic pathways. Literature for this review was identified through structured searches in PubMed, Scopus, Web of Science, and Google Scholar (2000–2025) using predefined keywords, with inclusion limited to peer-reviewed English studies on microalgal metabolites and apoptosis or neuroprotection. This review critically examines mechanistic, translational, and safety evidence, highlighting both therapeutic promise and current limitations, and proposes microalgae-derived metabolites as potential dual-action modulators for oncology and neurology.

## 1 Introduction

Programmed cell death (PCD) or apoptosis is a fundamental biological process that maintains cellular homeostasis *via* dysfunctional cells or repairing damage. It plays a key function in a varied range of physiological processes, like development, immune regulation, and tissue homeostasis. However, the dysregulation of the apoptosis procedure is an important feature of cancer, where evasion of cell death leads to tumor progression, metastasis, and resistance to treatment (Hanahan and Weinberg, 2011; Meehan and Vella, 2016). Thus, therapeutic strategies aimed at restoring or modulating apoptotic pathways in cancer cells have become a focus of cancer research. Conventional cancer therapies like chemotherapy and radiation have the intention to induce the apoptosis process in different tumor cells. However, these treatments are often non-selective, leading to substantial toxicity to normal tissues and the progression of resistance mechanisms (Carneiro and El-Deiry, 2020; Herr and Debatin, 2001). The resistance of different cancer cells to apoptosis is frequently facilitated by the inactivation of important apoptotic regulators, such as p53, and the overexpression of key anti-apoptotic proteins like Bcl-2 (Kollek et al., 2016; Opferman, 2007).

Aging serves as the predominant risk factor for the emergence of neurodegenerative diseases (NDs), including Parkinson’s disease (PD), Alzheimer’s disease (AD), and Huntington’s disease. These conditions are distinguished by a gradual and irreversible degeneration of selectively susceptible neurons, culminating in cognitive impairment, motor dysfunction, and behavioral anomalies (Hou et al., 2019; Azam et al., 2021). Two fundamental biological mechanisms that underlie these pathological alterations are apoptosis, a variant of programmed cellular demise, and neuroplasticity, which refers to the brain’s capability to reorganize synaptic connections in reaction to environmental, physiological, or pathological stimuli (Mattson and Arumugam, 2018; Mattson et al., 1999). Apoptosis is integral to neural development and homeostatic regulation. Nevertheless, in aging and neurodegenerative conditions, dysregulation of apoptotic pathways precipitates excessive neuronal loss (Fricker et al., 2018). Mitochondrial dysfunction, oxidative stress, DNA damage, and the stimulation of both extrinsic and intrinsic apoptotic pathways collectively converge to initiate the apoptotic cascade (Ashkenazi and Salvesen, 2014; Dillon and Green, 2016). In contrast, neuroplasticity, which includes synaptic remodeling, neurogenesis, and the reorganization of dendritic spines, is critical for cognitive processes such as learning, memory, and functional recovery (Karch and Goate, 2015). Nevertheless, the capacity for neuroplasticity diminishes with advancing age and is further compromised under conditions of neurodegenerative disorders (Morrison and Baxter, 2012; Hara et al., 2012). The strategic targeting of apoptosis and the enhancement of neuroplasticity emerge as promising and synergistic therapeutic approaches. Specifically, the inhibition of pro-apoptotic signaling pathways may serve to preserve neuronal viability, while concurrently, the promotion of neuroplasticity may facilitate the maintenance or restoration of neural circuitry and cognitive functions (Essa et al., 2012). Recent developments in the field of molecular neuroscience have elucidated a variety of modulatory factors, such as neurotrophic factors like brain-derived neurotrophic factor (*BDNF*)*,* transcriptional regulators including nuclear factor erythroid 2-related factor 2 (Nrf2) and cAMP-response element binding protein, signaling pathways, and small molecular entities that exert influence over both apoptotic processes and neuroplasticity (Long et al., 2021; Zarneshan et al., 2022).

Consequently, there is an increasing demand for new therapeutic approaches that can selectively target apoptotic pathways within cancer cells while minimizing harm to healthy tissues. In the current framework, microalgae have appeared as promising natural sources of therapeutic agents. Microalgae are unicellular, photosynthetic microorganisms found in freshwater and marine environments, classified primarily into groups such as Chlorophyta (green algae), Bacillariophyta (diatoms), Cyanobacteria (blue–green algae), and others. They produce diverse secondary metabolites with ecological and pharmacological significance (Morais Junior et al., 2020). These photosynthetic microorganisms synthesize a wide range of bioactive metabolites, like polysaccharides, carotenoids, and polyphenols, many of which have demonstrated anticancer properties *via* the modulation of main molecular pathways, including apoptosis (Matin et al., 2024). Notably, bioactive compounds from microalgae have demonstrated pro-apoptotic effects in various cancer types, including breast, lung, liver, cervical, and leukemia models (Roshan Cheragh and Mabudi, 2023; Sawasdee et al., 2023; Hamai-Amara et al., 2024; Silva et al., 2024; Al-Shajrawi et al., 2025). Microalgae, including cyanobacteria such as *Arthrospira platensis* Gomont (Oscillatoriaceae) (commonly known as spirulina), are diverse photosynthetic microorganisms that have gained significant consideration as promising sources of bioactive complexes with important anticancer impacts. These compounds include carotenoids, polyphenols, lipids, and polysaccharides, which have been shown to influence various molecular pathways involved in apoptosis (Saide et al., 2021; Martínez-Ruiz et al., 2022). Microalgae-derived bioactive compounds are particularly appealing because they can modulate both intrinsic and extrinsic apoptotic molecular pathways, which are critical in the selective elimination of different cancer cells while sparing normal cells (Bouyahya et al., 2024; Talero et al., 2015). Additionally, microalgae-based compounds have demonstrated the ability to improve the effectiveness of conservative therapies by overcoming mechanisms like multidrug resistance, which often hampers the success of cancer treatments (Bouyahya et al., 2024; Ahirwar et al., 2022). Also, microalgae have therapeutic potential effects against mitochondrial dysfunction, oxidative stress, inflammation, and neuronal cell death, key pathogenic features of neurodegeneration (Shah et al., 2016). Microalgae produce a diverse array of metabolites, including polyunsaturated fatty acids (PUFAs), carotenoids, peptides, and polysaccharides, many of which exhibit neuroprotective effects by controlling apoptosis and enhancing neuroplasticity (Rosic, 2025). For instance, fucoxanthin (FX) from *Phaeodactylum tricornutum* Bohlin (Phaeodactylaceae) has shown the ability to visualize amyloid-beta (Aβ) plaques and potentially inhibit aggregation in Alzheimer’s models (Lee et al., 2021). Similarly, extracts from *Desmodesmus* have demonstrated antioxidant and anti-apoptotic effects in PD models by modulating mitochondrial function, reducing reactive oxygen species (ROS), and upregulating neuroprotective pathways (Derya-Andeden et al., 2025). These findings suggest that microalgal bioactives may simultaneously regulate apoptotic pathways and promote neural regeneration, offering a dual-action approach for neurodegenerative disease therapy.

Despite the increasing scholarly interest in the therapeutic capabilities of natural products, bioactive compounds derived from microalgae represent an inadequately explored yet exceedingly promising category of agents that possess the ability to modulate essential biological processes, including apoptosis, neuroplasticity, and metabolic reprogramming, pathways that are frequently disrupted in both NDs and malignancies. Although individual research endeavors have elucidated the anticancer or neuroprotective properties of particular microalgal compounds, a comprehensive review that integrates these findings through the common paradigm of brain and tumor metabolism, emphasizing their dual regulatory functions in PCD and neurogenesis, is presently lacking. Furthermore, while the existing body of literature has separately examined the neuroprotective properties of microalgae in the context of NDs as well as their antitumor activities, no review has concurrently explored the intersecting molecular pathways influenced by these compounds across both areas of study. This is particularly salient in light of the growing acknowledgment of interconnected metabolic and signaling pathways, like oxidative stress, inflammation, mitochondrial impairment, PI3K/Akt/mTOR signaling cascades, and epigenetic modifications, that serve an important role in the etiology of both oncological and neurodegenerative disorders. Moreover, the brain-tumor axis, a nascent concept delineating the reciprocal interactions between cerebral functioning and tumor advancement, has yet to be thoroughly investigated within the framework of microalgal bioactive compounds. The capacity of these bioactive metabolites to function as pleiotropic modulators positions them as exemplary candidates for such dual-targeted therapeutic approaches; however, this promising avenue remains predominantly unexamined and devoid of contextualization within a cohesive biological paradigm. Given the swift proliferation of scholarly inquiry into both functional microalgal nutraceuticals and metabolism-oriented therapeutic interventions, an expeditious and integrative analysis is imperative. By connecting two principal pathological domains, oncology and neurodegeneration, this review aspires to address a significant lacuna in the existing literature and stimulate novel pathways in the formulation of multifunctional, metabolism-focused therapeutics derived from microalgal sources. While preclinical data are promising, variability in metabolite composition, limited bioavailability, and potential ecological or toxicological concerns from large-scale cultivation warrant careful risk–benefit evaluation.

## 2 Methodology

This review followed a structured narrative approach, ensuring comprehensive and transparent literature coverage. Searches were performed in PubMed, Scopus, Web of Science, and Google Scholar for publications from January 2000 to June 2025. Search strings were adapted for each database and combined Boolean operators with controlled vocabulary where available (e.g., MeSH in PubMed). The following search terms were applied in various Boolean combinations: “microalgae” OR “cyanobacteria” AND “apoptosis” OR “cell death” OR “neuroplasticity” OR “neurodegenerative diseases” OR “cancer” AND “bioactive compounds” OR “metabolites.” Only peer-reviewed articles in English were included. Eligible studies comprised *in vitro*, *in vivo*, or human research that investigated microalgae-derived compounds in the context of apoptosis or neuroprotection, with clearly reported experimental models and outcomes. Exclusion criteria included conference abstracts, review articles, and studies on non-microalgal organisms or lacking mechanistic relevance. All records were screened first by title/abstract, then by full-text review. Screening was conducted independently by the author to minimize selection bias, and disagreements on eligibility were resolved by rechecking the full text against the inclusion criteria. References of included studies were also examined to identify additional eligible articles. Studies were categorized as *in vitro*, *in vivo*, or clinical to facilitate comparison of evidence strength and relevance.

## 3 Overview of apoptosis

### 3.1 Definition and biological significance

Apoptosis or PCD, is a firmly regulated procedure crucial for tissue homeostasis and the elimination of damaged or abnormal cells. It occurs *via* two primary pathways: the intrinsic (mitochondrial) pathway, triggered by internal stress signals like oxidative stress and DNA damage, and the extrinsic pathway, initiated by external ligands binding to death receptors. Both pathways converge on the activation of caspases, a family of proteases that orchestrate cellular disassembly. Dysregulation of apoptosis is a hallmark of cancer, enabling tumor cells to evade death, resist therapy, and promote progression (Mustafa et al., 2024). Apoptosis is an essential physiological process that removes dysfunctional cells or damage, preserving tissue homeostasis and preventing tumor formation. It is strongly controlled by a balance between pro-apoptotic and anti-apoptotic signals. In cancer, this balance is often disrupted, leading to resistance to various cell death, metastasis, and uncontrolled proliferation. Elucidating the mechanisms of apoptosis in cancer is critical for identifying therapeutic targets and developing effective treatments.

### 3.2 Intrinsic apoptosis pathway

The intrinsic molecular pathway is started within the cell in response to internal stress signals like oxidative stress, oncogene activation, or DNA damage. Stress signals trigger pro-apoptotic members of the Bcl-2 family (e.g., Bak, Bax), causing mitochondrial outer membrane permeabilization (MOMP) and releasing cytochrome c into the cytoplasm (Green and Llambi, 2015). Furthermore, cytochrome c binds to apoptotic protease activating factor-1 (Apaf-1) to form the apoptosome, which subsequently activates caspase-9, leading to downstream activation of effector caspases (Elmore, 2007; D'Arcy, 2019). Also, stimulated effector caspases degrade cellular components, leading to characteristic apoptotic morphology, like cell shrinkage, chromatin condensation, and membrane blebbing (Kroemer et al., 2007).

### 3.3 Extrinsic apoptosis molecular pathway

**FIGURE 1:**
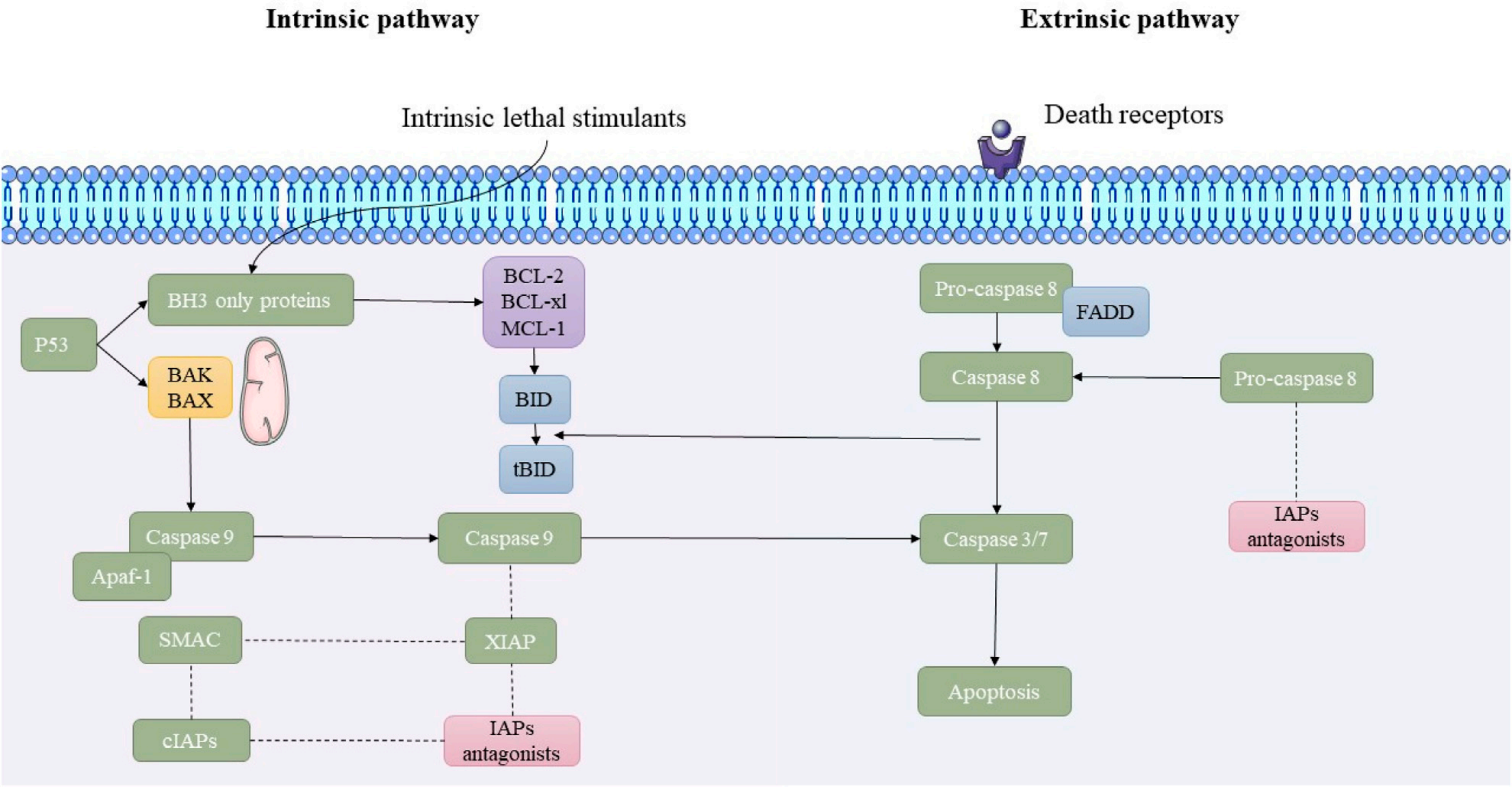
Overview of the intrinsic and extrinsic apoptosis pathways. This schematic illustrates the molecular mechanisms underlying the intrinsic and extrinsic pathways of apoptosis. The intrinsic pathway is initiated by lethal cellular stress, leading to p53-mediated activation of pro-apoptotic BAK/BAX proteins and mitochondrial outer membrane permeabilization (MOMP). This results in the release of cytochrome c and the activation of caspase-9 through apoptosome formation. In contrast, the extrinsic pathway is triggered by the binding of death ligands to death receptors, leading to FADD-mediated activation of caspase-8. Both pathways converge on the activation of executioner caspases-3/7, leading to apoptosis. Inhibitor of apoptosis proteins (IAPs) and their antagonists modulate the process.

This pathway is triggered by extracellular death ligands binding to their respective death receptors on the cell surface. Death ligands like Fas ligand, tumor necrosis factor-alpha (TNF-α), or TNF-related apoptosis-inducing ligand (TRAIL) bind to their receptors (e.g., Fas/CD95, TNF receptor, or TRAIL receptors) (Ashkenazi and Dixit, 1998). Furthermore, receptor activation recruit’s adaptor proteins like FADD, leading to the assembly of the DISC. This complex activates initiator caspases like caspase-8. Furthermore, caspase-8 directly stimulates effector caspases or cleaves Bid, a pro-apoptotic Bcl-2 protein, linking the extrinsic pathway to the mitochondrial pathway (Fulda and Debatin, 2006a; Fulda and Debatin, 2006b). The main molecular mechanisms underlying the extrinsic and intrinsic pathways of apoptosis are illustrated in Figure 1.

### 3.4 Apoptosis dysregulation in carcinogenesis

Cancer cells employ multiple molecular strategies to evade apoptosis. Upregulation of Bcl-XL, Bcl-2, or Mcl-1 prevents mitochondrial-mediated apoptosis (Adams and Cory, 2007; Labi et al., 2008). In addition, the downregulation of Bax, Bak, or Bid diminishes the cell’s ability to initiate apoptosis. Also, mutations or degradation of p53 impair apoptotic signaling (Vousden and Prives, 2009). Finally, pathways like PI3K/AKT and nuclear factor-kappa B (NF-κB) improve cell survival and inhibit apoptotic proteins (Manning and Cantley, 2007).

## 4 Microalgae and cancer hallmarks

**FIGURE 2:**
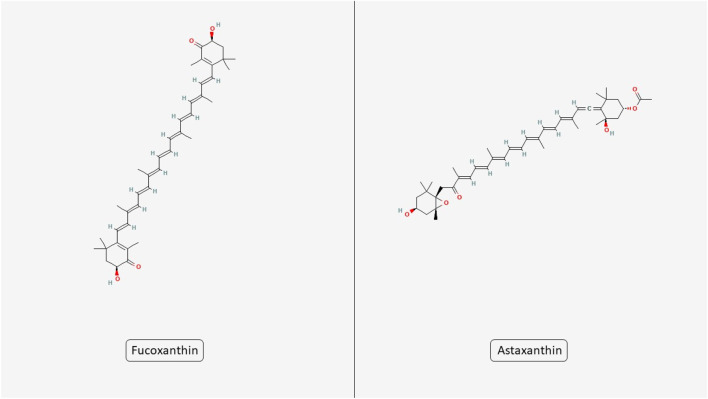
Chemical structures of microalgal carotenoids fucoxanthin and astaxanthin. The figure displays the molecular structures of two bioactive carotenoids derived from microalgae, fucoxanthin and astaxanthin.

Microalgae produce a diverse array of bioactive complexes that exert anticancer effects by targeting several hallmarks of cancer. These effects include promoting apoptosis, reducing oxidative stress, inhibiting cell proliferation, modulating inflammatory responses, and interfering with angiogenesis and metastasis. Such multitargeted actions make microalgae promising candidates for integrative cancer therapy approaches. In this section, we explore how specific metabolites derived from microalgae influence these critical cancer-related pathways. Also, the structures of two key microalgal carotenoids, FX and astaxanthin (AST), are shown in Figure 2.

### 4.1 Induction of apoptosis

Microalgae-derived bioactive metabolites can activate the apoptosis process in various cancer cells through numerous molecular mechanisms, including intrinsic pathway activation. These metabolites often act by controlling mitochondrial membrane potential and influencing the expression of apoptotic regulators such as Bcl-2, Bax, and cytochrome c, ultimately triggering caspase activation and cell death (Chiu et al., 2004). Metabolites such as FX and AST modify mitochondrial membrane potential, leading to caspase cascade stimulation and cytochrome c release (Ambati et al., 2014; Wu et al., 2019). These metabolites promote MOMP by activating pro-apoptotic Bcl-2 family proteins (e.g., Bak and Bax), which oligomerize and form pores in the mitochondrial membrane, leading to the release of cytochrome c into the cytosol and subsequent apoptosome formation (Chiu et al., 2004). Another mechanism is extrinsic pathway activation. Sulfated polysaccharides and specific peptides augment death receptor-mediated apoptosis *via* upregulating TRAIL and Fas (Cunha and Grenha, 2016). While the primary focus of this review is on apoptosis, it is worth noting that several microalgae-derived metabolites have also been implicated in modulating other forms of PCD, such as necroptosis and autophagy (Talero et al., 2015; Li et al., 2024). Although data on these pathways remain limited, early studies suggest that metabolites like FX and phycocyanin may influence autophagic signaling and cell survival mechanisms in pathologic conditions (Hu et al., 2024; Zhu et al., 2018). Further research is needed to clarify these effects and their potential therapeutic relevance. Evidence is predominantly from *in vitro* studies on single cancer cell lines, often reporting IC_50_ values without selectivity indices. While these data provide mechanistic insights, they should be interpreted cautiously until validated in *in vivo* models and clinical settings.

### 4.2 Regulation of oxidative stress

Microalgae are rich in antioxidants like phenolic compounds and carotenoids, which control ROS production. By preserving a balance in oxidative damage, these compounds protect normal cells while promoting ROS-induced apoptosis in the various cancer cells (Shanab et al., 2012). For instance, AST and phycocyanin act as potent free radical scavengers, reducing oxidative damage in normal cells while selectively increasing ROS levels in cancer cells to trigger apoptosis (Shin et al., 2022; Jiang et al., 2017; Liu et al., 2022). This dual action highlights the context-dependent antioxidant behavior of microalgae-derived metabolites, which may be exploited for targeted redox modulation in tumor environments.

### 4.3 The molecular effects of microalgae on apoptosis and neuroplasticity in aging and NDs

Microalgae are increasingly recognized for their unique bioactive metabolites that modulate critical cellular processes such as apoptosis and neuroplasticity, both of which are fundamental to the pathophysiology of aging and NDs. Aging brains experience reduced neuroplasticity and an imbalance in apoptotic signaling, leading to cognitive deficits and heightened vulnerability to neurodegeneration (Mattson and Arumugam, 2018). Microalgal metabolites, including carotenoids, PUFAs, peptides, and polysaccharides, can modulate these pathways through anti-inflammatory, antioxidant, anti-apoptotic, and neurotrophic mechanisms (Lu et al., 2025; Raposo et al., 2013). In addition, excessive or dysregulated apoptosis contributes significantly to neuronal loss in NDs such as AD and PD. Microalgal bioactives can attenuate apoptosis through upregulating BDNF, thus supporting synaptic plasticity and memory (Fakhri et al., 2018). Oxidative stress and chronic neuroinflammation are central to both apoptosis induction and neuroplasticity impairment. Microalgae-derived metabolites enhance antioxidant defenses in neuronal cells (Hu et al., 2018). Moreover, it downregulates inflammatory mediators and suppress NF-κB signaling, thereby indirectly preserving neuronal function and synaptic integrity (Gallego et al., 2022). These metabolites provide a protective environment that sustains neuroplasticity while preventing cell death. Notably, the apoptotic effects of microalgal metabolites are highly context-dependent: they can induce apoptosis in cancer cells while protecting neurons from apoptosis under stress. This selectivity reflects differences in redox status, mitochondrial dynamics, and signaling pathways between neuronal and tumor cells.

### 4.4 Inhibition of cell proliferation in different cancers

Bioactive metabolites from microalgae, such as eicosapentaenoic acid and phycocyanin, exhibit antiproliferative influences by arresting the cell cycle at specific checkpoints (Jiang et al., 2017) and downregulating oncogenic signaling pathways like PI3K/AKT, MAPK, and Wnt/β-catenin (Sawasdee et al., 2023). These effects are often achieved through the downregulation of cyclins (e.g., Cyclin D1) and CDKs, resulting in cell cycle arrest at G2/M or G0/G1 phases (Park et al., 2015). In parallel, microalgae metabolites may suppress oncogenic signaling cascades such as Wnt/β-catenin or PI3K/AKT, thereby blocking proliferation cues and supporting tumor suppression (Kavitha et al., 2013; Kowshik et al., 2019).

### 4.5 Anti-inflammatory properties in different cancers

Inflammation is a crucial driver of tumor progression. Microalgae-derived polysaccharides and carotenoids suppress pro-inflammatory markers such as IL-6 and TNF-α, and inhibit NF-κB signaling, reducing tumor-promoting inflammation (Ávila-Romá et al., 2021). In addition to reducing pro-inflammatory cytokines, microalgal polysaccharides and carotenoids have been shown to modulate immune responses by enhancing dendritic cell activity and natural killer (NK) cell cytotoxicity (Riccio and Lauritano, 2019; Santos et al., 1998). This suggests that microalgae not only suppress tumor-promoting inflammation but also contribute to the reactivation of anti-tumor immunity.

### 4.6 Anti-angiogenesis and metastasis

Compounds such as fucoidan and lutein inhibit angiogenesis by targeting VEGF and its associated pathways. Fucoidan is a sulfated polysaccharide mainly derived from brown algae, including certain marine microalgae in the class Phaeophyceae, such as those related to the genus Phaeodactylum (Bacillariophyceae). Lutein is a xanthophyll carotenoid predominantly produced by green microalgae in the class Chlorophyceae, such as *Chlorella* spp. and *Scenedesmus* spp. These metabolites have been widely studied for their antioxidant, anti-inflammatory, and anti-angiogenic properties, making them attractive candidates for therapeutic development (Chen et al., 2016). Additionally, microalgae extracts modulate matrix metalloproteinases (MMPs), limiting cancer cell invasion and metastasis (Khotimchenko et al., 2020). Fucoidan, for example, has been reported to downregulate MMP-2 and MMP-9, enzymes critical for extracellular matrix degradation and tumor invasion. In most studies, fucoidan was obtained *via* aqueous extraction from brown algae within the class Phaeophyceae, with microalgal sources such as *P. tricornutum* (Bacillariophyceae) also reported. Lutein, conversely, is typically extracted from green microalgae in the class Chlorophyceae, including *Chlorella* spp. and *Scenedesmus* spp., using organic solvents such as acetone or ethanol to ensure efficient carotenoid recovery.

Fucoidan, for example, has been reported to downregulate MMP-2 and MMP-9, enzymes critical for extracellular matrix degradation and tumor invasion (Reyes et al., 2020). By suppressing these targets, microalgae extracts may effectively hinder metastatic spread in aggressive cancers.

While numerous *in vitro* and *in vivo* studies have demonstrated the mechanistic potential of microalgal metabolites in modulating apoptosis and neuroplasticity, it is important to acknowledge that their translation to clinical efficacy depends strongly on bioavailability. Many key compounds, such as carotenoids (e.g., astaxanthin, fucoxanthin) and polyunsaturated fatty acids, display poor aqueous solubility, susceptibility to degradation, and limited absorption in the human gastrointestinal tract. These pharmacokinetic limitations can significantly reduce systemic exposure and tissue targeting, potentially diminishing the observed effects in human settings compared to experimental models (Ambati et al., 2014; Pangestuti and Kim, 2011; Yang et al., 2013). Strategies such as lipid-based carriers, nanoencapsulation, and formulation with bioenhancers have shown promise in improving stability, absorption, and targeted delivery, but further research is warranted before consistent therapeutic outcomes can be assured.

## 5 Microalgae as regulators of apoptosis and neuroplasticity in aging and NDs

**FIGURE 3:**
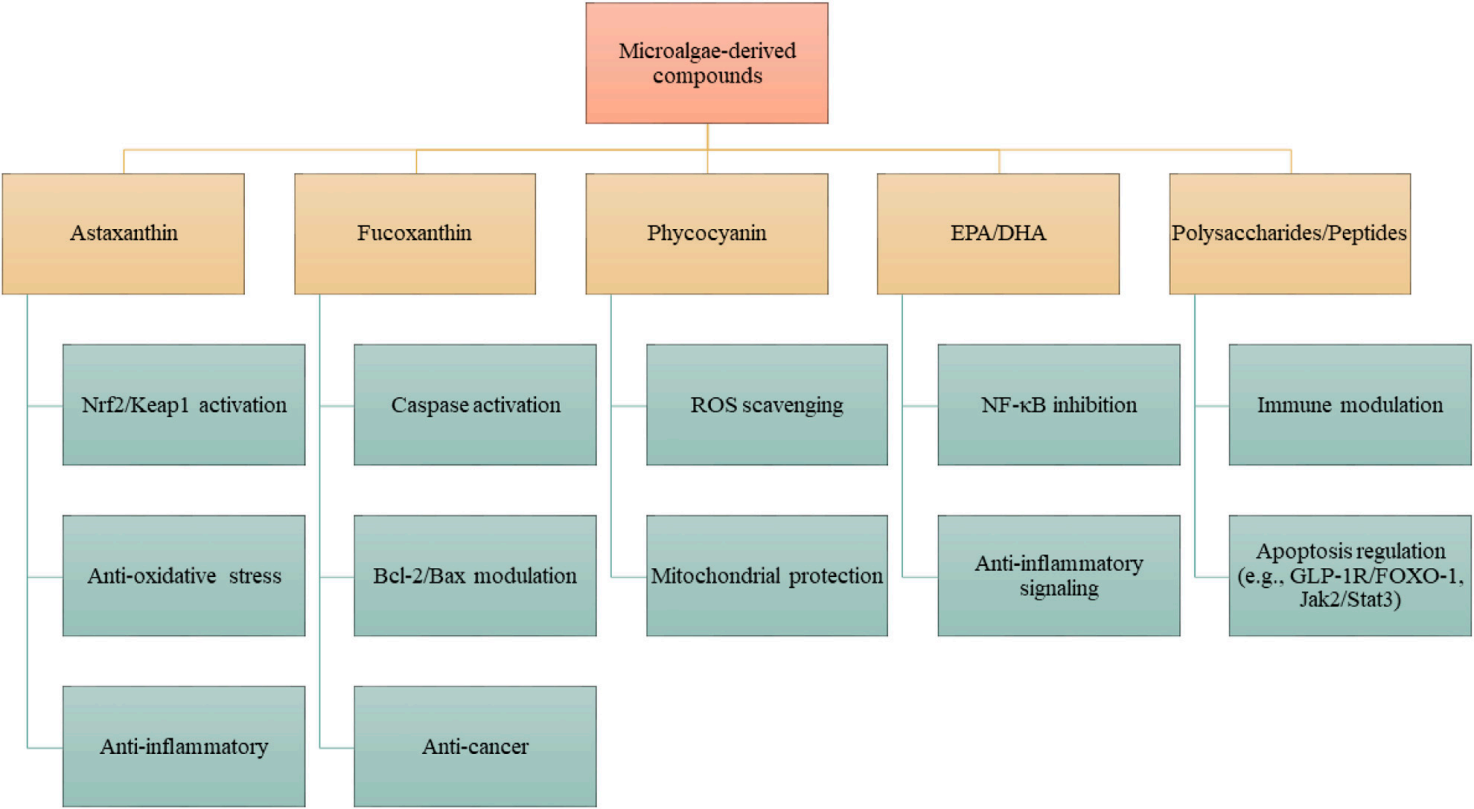
Proposed mechanisms of action of microalgae-derived compounds. Specific metabolites, including astaxanthin, fucoxanthin, phycocyanin, EPA/DHA, and polysaccharides/peptides, exert distinct pharmacological activities. These compounds modulate defined molecular pathways such as Nrf2/Keap1, NF-κB, Bcl-2/Bax, caspase cascades, and Jak2/Stat3, resulting in antioxidant, anti-inflammatory, anti-apoptotic, or neuroprotective effects. Effects are compound- and structure-dependent, rather than universal to all microalgae.

NDs are distinguished by the progressive degeneration of neurons, metabolic dysregulation, and compromised neuroplasticity, accompanied by a scarcity of effective therapeutic interventions. Recent findings underscore the interplay of disrupted cerebral metabolism, oxidative stress, and aberrant apoptosis as pivotal mechanisms that propel the advancement of these diseases. Bioactive metabolites derived from microalgae present considerable neuroprotective potential by modulating these deleterious pathways (Figure 3). It is important to note that the apoptotic response to microalgal bioactives is highly context-dependent: in neurons exposed to oxidative or inflammatory stress, they act mainly through anti-apoptotic and cytoprotective mechanisms, whereas in cancer cells, they frequently promote apoptosis through mitochondrial disruption and caspase activation.

It is also imperative to elucidate the effects of microalgal compounds on cerebral metabolism, apoptotic processes, and neurogenesis to formulate innovative therapeutic approaches. Notwithstanding the increasing scholarly attention, a thorough synthesis of the mechanisms through which these natural agents confer neuroprotective effects is conspicuously absent.

### 5.1 Aging

Aging is frequently correlated with cognitive deterioration and memory dysfunction, with inflammatory markers and oxidative stress indicators being involved in this intrinsic process. Extracts derived from microalgae serve as a natural reservoir of various bioactive metabolites that mitigate oxidative stress and inflammation, thereby presenting a novel dietary strategy to alleviate age-associated cognitive decline and memory dysfunction. A 24-week investigation assessed the ramifications of supplementation with *P. tricornutum* (Pt) microalgae on cognitive performance, mood, stress levels, and inflammatory responses in a cohort of 66 elderly subjects (aged 55–75 years old) exhibiting age-associated memory impairment. Subjects were administered either a placebo or 550 mg of Pt extract, which included FX and fatty acids. Although no statistically substantial changes were seen between the groups concerning primary cognitive outcomes, within-group analyses indicated that the Pt group exhibited enhancements in reaction time and delayed word recall. Furthermore, the Pt group reported a reduction in perceived stress and a noteworthy decrease in the inflammatory marker hs-CRP (Goodbody et al., 2025) (Table 1). Another investigation explored the repercussions of both virgin and aged polyvinyl chloride (PVC) microplastics (MPs) on *Chlorella pyrenoidosa* H.Chick (Chlorellaceae), a species of freshwater microalga. Over a duration of 96 h, both variants of PVC MPs impeded algal proliferation, with aged PVC exhibiting heightened toxicity (up to 44.72% inhibition of growth). The aged MPs resulted in more pronounced decreases in intracellular constituents, chlorophyll a levels, and photosynthetic efficiency (Fv/Fm), signifying a more profound disturbance of the photosynthetic process. Furthermore, aged PVC enhanced nitrogen assimilation and facilitated the synthesis of humic acid-like metabolites. The identified toxicity was intricately associated with increased intracellular oxidative stress within the algal cells (Xu Y. et al., 2024).

**TABLE 1:** Summary of studies on microalgal bioactives in aging and neurodegenerative models. Compounds are specified as purified metabolites, enriched fractions, or whole biomass extracts.

Condition/Model	Compound/Intervention	Source organism	Key outcomes	References
Aging (human, i*n vivo*)	*Phaeodactylum tricornutum* extract (550 mg/day; 4.4 mg fucoxanthin, PUFAs, SFAs)	*P. tricornutum*	Improved Stroop reaction time and word recall; reduced stress; decreased hs-CRP. Mechanisms: anti-inflammatory (↓ CRP), antioxidant via fucoxanthin, neuroprotection.	Goodbody et al. (2025)
Aging (human, i*n vivo*)	Whole biomass and β-1,3-glucan–rich supernatant	*P. tricornutum*	Lower plasma IL-6 and fecal zonulin; improved sit-to-stand performance; reduced ω-6/ω-3 and AA/EPA ratios. Mechanisms: anti-inflammatory, antioxidant, improved gut barrier integrity.	Stiefvatter et al. (2022)
Aging-related diabetes (mice)	Low-molecular-weight polysaccharide (CPP)	*Chlorella pyrenoidosa*	Increased SOD, CAT, GPX in liver/brain; enhanced insulin secretion; tissue repair. Mechanisms: GLP-1R/FOXO-1 activation; IL-6R/FOXO-1 inhibition; antioxidant defense.	Qiu et al. (2022)
Aging (rats, D-galactose model)	Astaxanthin-rich biomass and fractions	*Haematococcus pluvialis*	Restored liver function; ↑ CAT, GST, MPO; ↓ IL-6, NF-κB; improved histology. Mechanism: Nrf2 activation; suppression of NF-κB and IL-6.	El-Baz et al. (2018)
Aging (human, i*n vivo*)	*P. tricornutum* extract (1,100 mg/day; 8.8 mg fucoxanthin)	*P. tricornutum*	Improved memory, attention, executive function, insulin sensitivity, and sleep. Mechanisms: fucoxanthin antioxidant/anti-inflammatory, modulation of BDNF and insulin pathways.	Yoo et al. (2024)
Alzheimer’s disease (C. elegans model)	Phytoene (purified) and phytoene-rich extracts	*Chlorella sorokiniana*, *Dunaliella bardawil*	Extended lifespan; protection from oxidative stress; reduced Aβ42 toxicity. Mechanisms: antioxidant activity; proteotoxicity modulation; longevity pathways.	Morón-Ortiz et al. (2024)
Alzheimer’s disease (*in vitro*)	LTB:RAGE vaccine candidate	*Schizochytrium* sp.	Stable, thermostable vaccine candidate; prevented Aβ–RAGE interaction.	Ortega-Berlanga et al. (2018)
Alzheimer’s disease (mice, i*n vitro* imaging)	Fucoxanthin (purified metabolite)	*P. tricornutum*	Detected Aβ plaques; colocalized with thioflavin S. Mechanism: fucoxanthin binds Aβ, enables fluorescence visualization.	Lee et al. (2021)
Alzheimer’s disease (*in vitro*)	Crude extracts (72 strains tested)	Chlorophyceae, Eustigmatophyceae	27/72 extracts inhibited QC; activity varied by growth phase. Mechanism: inhibition of QC prevents Aβ oligomer formation.	Krause-Hielscher et al. (2015)
Parkinson’s disease (SH-SY5Y cells)	Methanol extract (*Desmodesmus* sp., DaMe)	*Desmodesmus* sp.	Reduced ROS/DNA damage; ↑ mitochondrial potential; regulated PD-related apoptosis genes. Mechanism: antioxidant, mitochondrial protection.	Derya-Andeden et al. (2025)
Parkinson’s disease (SH-SY5Y cells)	Octapeptide IEC (Ile-Ile-Ala-Val-Glu-Ala-Gly-Cys)	*Isochrysis zhanjiangensis*	Reduced α-syn aggregation; ↑ HO-1, GPX; improved Bcl-2/Bax ratio; ↓ caspases, p53. Mechanisms: Nrf2 activation, anti-apoptotic signaling, protein aggregation suppression.	Liu et al. (2024)

A research investigation examined the impact of tire wear particles (TWPs) on the microalga *P. tricornutum* under diverse concentrations and aging conditions. Low values of TWPs (0.6 and 3 mg/L) facilitated algal proliferation, whereas elevated concentrations (15 and 75 mg/L) impeded it. Aged TWPs elicited more pronounced toxic impacts compared with virgin TWPs, encompassing diminished chlorophyll a levels, oxidative stress, and modified photosynthetic efficiency. Metabolomic analysis indicated that aged TWPs instigated more substantial metabolic disturbances. The augmented toxicity associated with aged TWPs was attributed to surface alterations and the increased release of hazardous additives. These results underscore the significance of accounting for the aging process when evaluating the environmental hazards posed by TWPs (Lv et al., 2024). A preliminary investigation examined the impact of *P. tricornutum* (PT) supplements, characterized by high concentrations of eicosapentaenoic acid (EPA), carotenoids, vitamins, and β-glucans, on a cohort of 19 physically healthy elderly individuals over a duration of 2 weeks. The participants were administered either whole PT (A), a β-1,3-glucan-enriched supernatant (SupB), a synergistic combination (A + SupB), or a control product. The synergistic combination (A + SupB) yielded a statistically significant reduction in plasma interleukin-6 (IL-6) levels and enhanced mobility, as evidenced by improvements in the sit-to-stand test and gait speed trends, in comparison to the control group. While no discernible differences in fatty acid profiles were noted among the groups, the A + SupB cohort exhibited reductions in the omega-6/omega-3 and arachidonic acid/EPA ratios. Furthermore, SupB was associated with a decrease in fecal zonulin concentrations, suggesting an enhancement in gut barrier integrity (Stiefvatter et al., 2022).

Another scholarly investigation analyzed the influence of microalgal biofouling on the aging of microplastics (MPs) and their capacity to adsorb organic micropollutants. Microplastics composed of polyvinyl chloride (PVC), polyethylene (PE), and polyamide (PA) were subjected to microalgal exposure in photobioreactors alongside river water microbial communities over a period of 30 days. The presence of algal biofouling resulted in considerably more pronounced surface degradation (such as cracks, pits, and polymer scission) and aging compared to that induced by river microbial biofouling. This phenomenon led to an augmentation of the adsorption capacity of PVC and PE for bisphenol analogues and parabens by factors of up to 6.72 and 8.72, respectively. The adsorption process was found to be dependent on pH, endothermic in nature, and facilitated by hydrogen bonding interactions. Conversely, the aging process associated with algal exposure diminished the adsorption capacity of PA due to the degradation of amide functional groups. Collectively, algal-aged microplastics exhibited a significantly enhanced potential for pollutant adsorption in comparison to both virgin and river-aged microplastics (Kiki et al., 2022). A research investigation concerning aging-associated diabetic murine models demonstrated that *C. pyrenoidosa* polysaccharide (CPP) exhibits significant hypoglycemic and antioxidant properties. CPP was observed to enhance the action of antioxidant enzymes like CAT, superoxide dismutase (SOD), and glutathione peroxidase (GPX) within hepatic and cerebral tissues, augment insulin secretion, and facilitate the repair of tissue damage across the brain, liver, pancreas, and jejunum. These advantageous effects were correlated with the activation of the GLP-1 receptor/FOXO-1 signaling pathway and the inhibition of the IL-6 receptor/FOXO-1 signaling pathway. Metabolomic analyses indicated that CPP mitigates the effects of aging-related diabetes through the enhancement of insulin levels and the reduction of oxidative stress, potentially through the modulation of phenylpyruvic acid (Qiu et al., 2022).

The investigation scrutinized the protective properties of zeaxanthin heneicosylate (ZH), a carotenoid ester derived from *Dunaliella salina*, against cardiac dysfunction in rats instigated by d-galactose (d-GAL). The administration of d-GAL resulted in substantial cardiac impairment, as evidenced by electrocardiogram alterations and heightened indicators of myocardial injury and inflammation. The intervention with ZH (250 μg/kg over a period of 28 days) yielded enhancements in cardiac functionality by reinstating antioxidant enzyme activity, alleviating inflammation, and ameliorating tissue injury. The advantageous impacts of ZH were related to the augmented expression of retinoid receptor alpha (RAR-α), corroborated by molecular docking analyses (El-Baz et al., 2019). Another work examined the hepatoprotective properties of *Haematococcus pluvialis* Flotow (Haematococcaceae), which is abundant in AST, against hepatic injury induced by D-galactose (D-Gal) in a rat model of aging. The administration of D-Gal resulted in hepatic dysfunction, oxidative stress, and inflammatory responses, as evidenced by elevated levels of liver enzymes, glutathione S-transferase, CAT, myeloperoxidase, along with increased IL-6 and NF-κB, in conjunction with diminished expression of Nrf2. Treatment with AST-enriched H. pluvialis effectively reinstated hepatic function, attenuated both oxidative and inflammatory biomarkers, and enhanced the expression of Nrf2. Histological evaluations corroborated these enhancements. Additionally, molecular docking analyses revealed a significant binding affinity of trans-AST to Nrf2, as well as a moderate affinity to NF-κB and IL-6, thereby substantiating its potential therapeutic applications (El-Baz et al., 2018).

An investigation examined the impact of PT extract, which is abundant in FX, on cognitive functions in healthy, physically active older adults who reported experiencing memory decline. A total of forty-three subjects were randomly allocated to receive either a placebo or a daily dosage of 1,100 mg of PT (equivalent to 8.8 mg of FX) over a period of 12 weeks. The supplementation of FX resulted in statistically significant or nearly significant enhancements in various cognitive assessments, encompassing word recall, reaction time, and attention-related tasks. Although the majority of enhancements were observed longitudinally rather than intergroup, FX consistently facilitated superior cognitive performance from baseline measurements, indicating prospective cognitive advantages (Yoo et al., 2024).

### 5.2 Alzheimer’s disease

Phytoene represents a colorless carotenoid that is abundantly sourced from dietary metabolites and serves as a precursor for the biosynthesis of various carotenoids. Despite its prevalent presence in elevated concentrations within diverse tissues, phytoene is predominantly regarded as lacking physiological function. Research conducted utilizing *Caenorhabditis elegans* has elucidated that phytoene possesses anti-aging characteristics, providing protection against oxidative stress and the neurotoxicity associated with amyloid-β42, while simultaneously prolonging lifespan. Extracts derived from *Chlorella sorokiniana* and *Dunaliella bardawil*, both of which are abundant in phytoene, exhibited analogous protective effects. These results substantiate phytoene’s classification as a bioactive metabolite that exerts advantageous influences on aging and longevity (Morón-Ortiz et al., 2024). The receptor for advanced glycation end products (RAGE), which mediates the transport of Aβ into the central nervous system and plays a significant role in the progression of AD, represents a viable target for therapeutic intervention. A research investigation employed the Algevir expression system in Schizochytrium sp. microalgae to generate a candidate vaccine, identified as LTB: RAGE. This inducible expression system attained production levels of up to 380 μg/g of fresh biomass within a timeframe of 48 h. The vaccine exhibited retained antigenic properties and demonstrated stability at temperatures reaching up to 60 °C, thereby underscoring the potential of Schizochytrium sp. as an advantageous platform for the progress of thermostable vaccines against AD (Ortega-Berlanga et al., 2018).

Glutaminyl cyclase (QC) has been related to a variety of pathological conditions, including AD, thereby rendering it a significant candidate for therapeutic intervention. Algae, recognized for their ability to synthesize a wide array of bioactive substances, were evaluated through a “Reverse Metabolomics” methodology coupled with Activity-correlation Analysis (AcorA) to pinpoint potential QC inhibitors. The investigation identified three sulfolipid metabolites derived from microalgae that exhibited pronounced QC inhibition, 81% and 76% at concentrations of 0.25 mg/mL and 0.025 mg/mL, respectively, underscoring their promise in enzyme-targeted therapeutic strategies (Hielscher-Michael et al., 2016). FX, a luminescent metabolite derived from the microalga *P. tricornutum*, has been demonstrated to effectively identify Aβ aggregates in murine models of AD. With optimal excitation and emission characteristics, FX facilitated the visualization of Aβ plaques within the cerebral tissues of APP/PS1 and 5×FAD murine models. Histological staining revealed that FX-positive areas exhibited colocalization with thioflavin S staining, thereby corroborating its efficacy in the detection of Aβ aggregates, positioning it as a promising instrument for the diagnosis of AD (Lee et al., 2021). QC is integral to the pathophysiology of AD, as it catalyzes the transformation of amyloid-β peptides into neurotoxic, degradation-resistant pyroglutamated derivatives that facilitate the formation of harmful oligomers. In an effort to identify natural inhibitors of QC, algal species from the Chlorophyceae and Eustigmatophyceae classes were cultivated and subjected to a rigorous screening process. Among the 72 algal extracts evaluated, 27 exhibited notable QC inhibitory activity. Subsequent fractionation utilizing Sephadex G-15 chromatography corroborated the existence of compounds that inhibit QC, thereby underscoring the potential of algae as a valuable source of natural QC inhibitors for the progress of therapeutic strategies against AD (Krause-Hielscher et al., 2015).

### 5.3 Parkinson’s disease

A research investigation examined the neuroprotective properties of a methanol extract derived from *Desmodesmus arthrodesmiformis* EM13 (DaMe) in the context of PD utilizing *in vitro* methodologies. Human SH-SY5Y neuroblastoma cells were subjected to pretreatment with DaMe prior to their exposure to 6-hydroxydopamine (6-OHDA), a neurotoxin associated with PD pathophysiology. The application of DaMe at concentrations ranging from 100 to 500 μg/mL resulted in a significant reduction of ROS, total oxidant status, and oxidative DNA damage, concomitantly enhancing total antioxidant capacity and mitochondrial membrane potential (ΔΨm). Furthermore, DaMe exhibited modulation of gene expression associated with PD and apoptotic pathways, thereby suggesting its potential utility as a protective agent against mitochondrial impairment in the context of PD (Derya-Andeden et al., 2025). Another work has elucidated the octapeptide IEC (Ile-Ile-Ala-Val-Glu-Ala-Gly-Cys) derived from the microalgae *Isochrysis zhanjiangensis* Dong and Huang (Isochrysidaceae), which exhibited notable antioxidant and neuroprotective properties. Molecular dynamics simulations have demonstrated a stable interaction between IEC and α-synuclein, a protein implicated in the pathophysiology of PD, thereby indicating its prospective ability to impede protein aggregation. In cellular models of PD induced by 6-OHDA in SH-SY5Y cells, IEC diminished oxidative stress through the stimulation of the Nrf2 signaling pathway, resulting in the upregulation of antioxidant enzymes such as GPX and HO-1. Furthermore, IEC exhibited an inhibitory effect on apoptosis by enhancing the Bcl-2/Bax ratio, attenuating the caspase cascade, and modulating the Jak2/Stat3 and p53 signaling pathways. These findings substantiate the role of IEC in mitigating oxidative stress and neurotoxicity associated with PD (Liu et al., 2024). Overall, bioactive compounds from microalgae demonstrate substantial potential as modulators of cerebral metabolism, apoptosis, and neuroplasticity within the context of NDs. Their capacity to simultaneously target multiple pathological pathways affords a distinctive advantage over traditional single-target therapeutic modalities. By amalgamating insights from cellular, molecular, and metabolic frameworks, these compounds may facilitate the development of pioneering, nature-derived therapeutic interventions. While these findings underscore the neuroprotective potential of microalgal metabolites, limitations include reliance on preclinical models, variability in metabolite composition, and unknown long-term safety in humans. Controlled clinical trials are needed before definitive therapeutic claims can be made.

## 6 Microalgae and molecular apoptotic pathways in cancer

**FIGURE 4:**
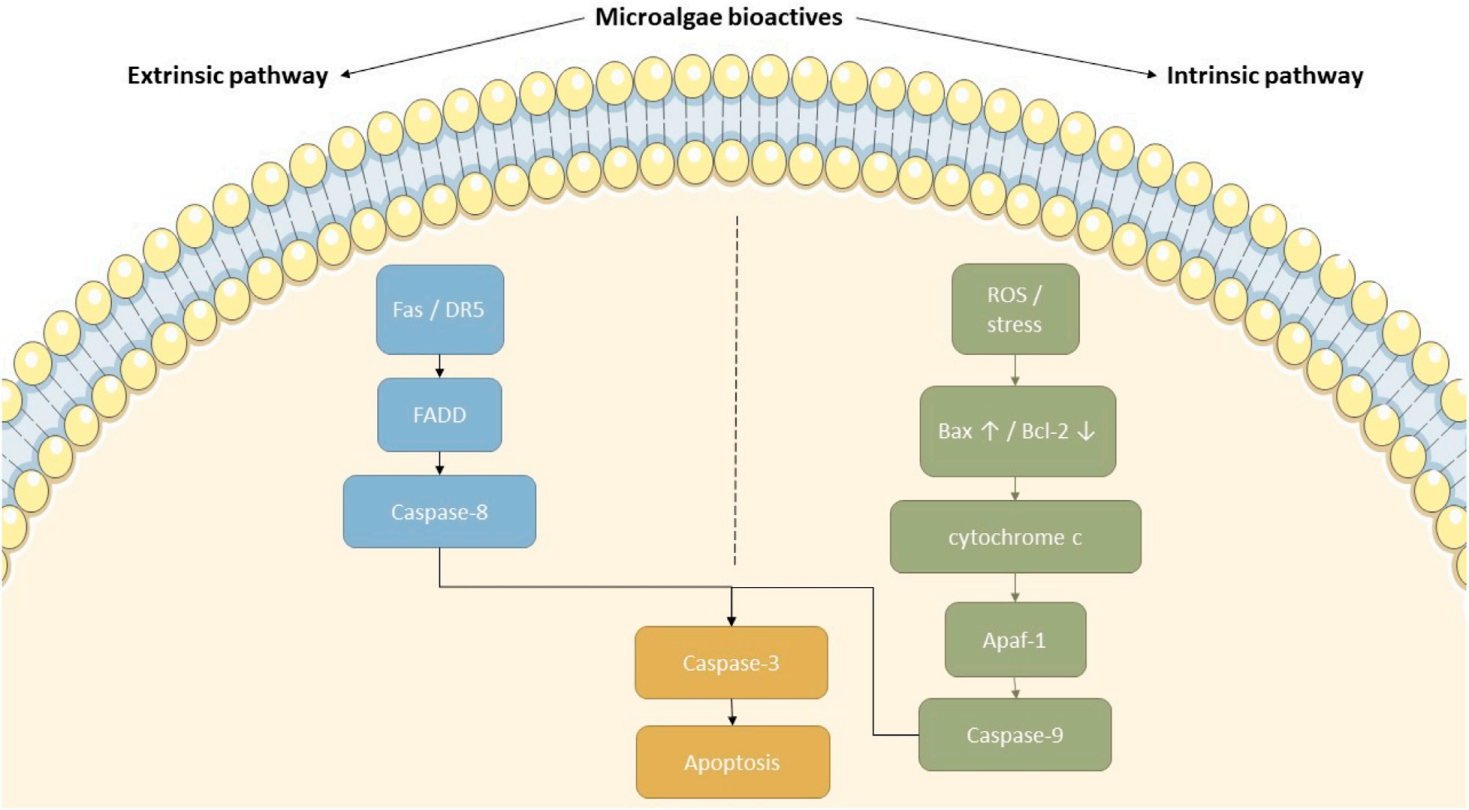
Proposed mechanisms of apoptosis induction by microalgal bioactive compounds. This diagram depicts how microalgal-derived compounds can activate both the intrinsic and extrinsic apoptotic pathways. In the extrinsic pathway, ligands binding to Fas or DR5 trigger FADD and caspase-8 activation. On the intrinsic side, ROS and cellular stress upregulate Bax and downregulate Bcl-2, facilitating cytochrome c release and caspase-9 activation through Apaf-1. Both cascades ultimately converge on caspase-3 activation, resulting in apoptosis. This figure highlights the therapeutic potential of microalgal compounds in targeting cancer cell survival mechanisms.

While the previous section introduced the role of microalgae in modulating multiple cancer hallmarks, including apoptosis, this section focuses specifically on the detailed molecular mechanisms by which microalgal compounds activate extrinsic and intrinsic apoptotic pathways in cancer cells (Figure 4). This section presents mechanistic insights into how microalgal compounds influence each of these apoptotic cascades in various cancer models. For clarity, findings are presented by evidence type: *in vitro* cell-based studies, *in vivo* animal models, and early clinical data where available.

Recent studies confirm that phycocyanin from *Spirulina platensis* induces apoptosis in lung cancer cells by disrupting mitochondrial membrane potential and enhancing ROS generation (Jiang et al., 2017). A study investigated the characteristics and anticancer properties of extracellular polysaccharide (EPS) derived from the DHA-producing microalga Crypthecodinium sp. SUN. The study found that EPS from *C. sp*. SUN strongly suppressed the proliferation and migration of LA795 lung adenocarcinoma cells, while the apoptosis rate fell in a concentration-dependent way, *C. sp*. SUN EPS dramatically reduced SOD activity by 76%, ROS by more than 50%, and CAT activity by 34%, showing that EPS may prevent tumor cell development rather than destroy tumor cells. Furthermore, by downregulating the expression of cyclin D1 and adhesion proteins in LA795 cells, *C. sp*. SUN EPS inhibited cell growth. Without influencing the naked mice’s normal body weight, *in vivo* tests showed that *C. sp*. SUN EPS reduced the progress of lung adenocarcinoma tumors (Ye et al., 2023) (Table 2). Another study found that surface-engineered microalgae *Chlorella vulgaris* Beijerinck (Chlorellaceae) improved with metal-organic framework (MOF) nanoparticles (denoted Chl-MOF) are effective for immunotherapy and photo-sonodynamic treatment. The results showed that Chl-MOF formed ROS during ultrasonic and laser, which heightened the photo-sonodynamic properties and improved tumor cell death. In addition, because of Chl’s immunomodulatory effects, Chl-MOF augmented NK cell cytotoxicity, amplified dendritic cell antigen-presenting ability, reversed the establishment of an immunosuppressive tumor microenvironment, and induced a relatively strong antitumor immune response. Chl-MOF diminished breast cancer size by 88.8% *in vivo* and *in vitro* with a mixture of immunotherapy and photo-sonodynamic treatment. Combined Chl and MOF offer a practical platform for cancer theranostic applications (Xu C. et al., 2024). However, these results derive from single-cell line models, often without selectivity indices, and thus require cautious interpretation.

**TABLE 2:** The beneficial effects of microalgae on molecular pathways related to apoptosis in various cancers.

Type of study	Type of cancer	Compound	Results	References
*In vitro*/*in vivo*	Lung adenocarcinoma cells	Extracellular polysaccharide from DHA-producing microalga *Crypthecodinium* sp. SUN	Inhibited the proliferation and migration, and apoptosis rate decreased in a concentration-dependent manner	Ye et al. (2023)
*In vitro*/*in vivo*	Breast cancer	Surface-engineered microalgae *Chlorella vulgaris* modified with metal‒organic framework nanoparticles	Increased natural killer cell cytotoxic activity, increased dendritic cell antigen-presenting ability, reversed the establishment of an immunosuppressive tumor microenvironment, enhancing tumor cell apoptosis	Xu et al. (2024b)
*In vitro*	Cervical and mammary carcinoma cell lines HeLa and MCF-7	Green microalga *Coelastrella* sp. BGV	Induce cancer cell death *via* the apoptosis pathway	Toshkova-Yotova et al. (2024)
*In vitro*	Breast carcinoma	Utilized *spirulina platensis* extract	Evaluated levels of Caspase - 3, −8 and – 9, induced apoptosis	Afzali et al. (2024)
*In vitro*	Cell lines of lung cancer (A549), breast cancer (MCF7), cervical cancer (Hela), cholangiocarcinoma (CCA; KKU213A), hepatocellular carcinoma (Huh7)	Chlorella sp., Sargassum spp., and Spirulina sp.	Induced cancer cell death *via* inhibition of AKT/mTOR pathway	Sawasdee et al. (2023)
*In vitro*	Human leukemia cell line K562	Marine microalga *Skeletonema marinoi*	Increase of apoptosis and the proapoptotic protein Bax and a decrease of the antiapoptotic protein Bcl-2	Ciarcia et al. (2022)
*In vitro*	Breast cancer cell	*Haematococcus pluvialis*	Suppressed BC cell growth, inhibited migration and invasion and induced apoptosis	Alateyah et al. (2022)
*In vivo*	Glioblastoma	Spirulina microalgae	Inhibited C6 and U87 cell proliferation and induced cell death	Arab et al. (2021)
*In vivo*	Advanced murine hepatocellular carcinoma	Spirulina	Increased endogenous antioxidant capacity, apoptosis (Bax) and p53	Mahmoud et al. (2021)
*In vitro*	Human Fetal Osteoblast Cells and osteosarcoma cells	*Schizochytrium* sp.	Induced apoptosis	Sahin (2021)
*In vitro*	Cell lines of human cancers, such as lung, skin melanoma, colorectal, breast and prostatic cancers	Microalgae of the Cuatro Cienegas Basin	Induced apoptosis	Tavares-Carreón et al. (2020)
*In vitro*	VDW cells	*Synechococcus* sp.	Induced the apoptotic pathway and activities of caspases-3, -8 and -9	Suttisuwan et al. (2019)
*In vitro*	MDA-MB-231, MCF-7, Hep-G2, and A-549 cell liens	*Picochlorum* sp. RCC486	Increased caspase-3 activity	Abolhasani et al. (2018)
*In vitro*	Murine L5178Y-R lymphoma cell line	Nuevo Leon regional *Chlorella sorokiniana*	Induced apoptosis	Reyna-Martinez et al. (2018)
*In vitro*	Cell lines of A375, Hs578T and HeLa	*Bo tryidiopsidaceae* sp	inhibited proliferation, invasion, and migration, and induced apoptosis	Suh et al. (2017a)
*In vitro*	HeLa, A375 and Hs578T	*Chloromonas* sp.	Inhibited cell growth and induced apoptosis	Suh et al. (2017b)
*In vitro*/*in vivo*	Cell lines of human cancers, such as stomach, lung, prostate, breast, pancreatic cancers, osteosarcoma	Marine microalgal	Increased apoptosis	Somasekharan et al. (2016)
*In vitro*	Human lung (A549) and breast cancer (MCF-7) cells	Euglena tuba (ETME)	Induced apoptosis, suppressing the activation of ERK1/2, JNK, P38 mitogen-activated protein kinase pathways	Panja et al. (2016)
*In vitro*	human lung carcinoma A- 549 cell lines	Spirulina	Increased apoptosis	Jubie et al. (2015)
*In vitro*	MDA-MB-231 cells	Calcitrans and N. oculata	Increased apoptosis	Goh et al. (2014)
*In vitro*	Hepatocarcimona (HepG2) cells	Navicula incerta	Increased apoptosis	Kim et al. (2014)
*In vitro*/*in vivo*	MDA-MB-231 cells	Spirulina	increased Bax and decreased Bcl-2 expression, induction of apoptosis	Ouhtit et al. (2014)
*In vitro*	Human breast cell lines	*Chaetoceros calcitrans* (UPMAAHU10)	Induced apoptosis and activating caspase 7	Ebrahimi et al. (2013)

In another study, the chemical properties, proapoptotic, and anticancer actions of EPS isolated from the green microalga *Coelastrella* sp. (Scenedesmaceae) BGV was examined. To separate EPS from the culture medium, a rapid and easy cold ethanol precipitation technique was adopted. The separated EPS sample has three carbohydrate fractions with varying molecular weights (11.5 × 104 Da, 30.7 × 104 Da, and 72.4 × 104 Da, respectively) and 7.14 (w/w%) protein content. The anticancer potential of the resulting EPS was evaluated *in vitro* utilizing cell lines from malignant and non-cancerous sources. The findings revealed that EPS lowered the viability of the mammary and cervical carcinoma cell lines MCF-7 and HeLa, but the control non-cancer cell lines HaCaT and BALB/3T3 were less impacted. The data revealed that the EPS treatment resulted in large increases in the sub-G1 cell population while decreasing the percentages of cells in the G1, S, and G2-M phases as compared with the control. The capacity of EPS to cause cancer cell death through the apoptotic pathway was amply confirmed by the fluorescence microscopy investigations conducted utilizing three distinct staining techniques. Additionally, EPS-treated HeLa cancer cells showed a different design and strength of immunocytochemical staining for the proteins bcl2, p53, and Ki67, which are related to proliferation and apoptosis, in comparison to the untreated controls (Toshkova-Yotova et al., 2024). Using a biological synthesis technique, a study investigated the synthesis, anticancer, and characterization characteristics of silver chloride nanoparticles (AgCLNPs) as a potential treatment option for breast cancer cells. AgCLNPs-SP’s apoptotic and anticancer characteristics were investigated in detail. The findings showed that AgCLNPs-SP, which are mostly silver and chlorine, have a spherical shape and range in size from 40 to 70 nm. The MTT Assay was used to determine AgCLNP-SP’s dose-dependent response against MDA-MB231 cells, yielding an IC50 value of 34 μg/mL. Additionally, MDA-MB231 cells showed a considerable percentage of early apoptosis (43.67%). The upregulation of CAD, P53, and Bax gene mRNA expression levels and the downregulation of bcl2 supported this apoptotic process. Furthermore, AgCLNPs-SP-treated MDA_MB231 cancer cells indicated increased ROS generation and Caspase-3, -8, and -9 (Afzali et al., 2024). While animal models confirm efficacy, translation to human therapy remains untested.

Alternative and efficient therapies are necessary since drug resistance and recurrence following existing conventional treatments are common. Crude polysaccharide and ethanolic extracts from Chlorella sp., Sargassum spp., and Spirulina sp. were studied for their anti-cancer properties against cell lines of five of the most common cancers: cervical cancer (Hela), breast cancer (MCF7), lung cancer (A549), cholangiocarcinoma (CCA; KKU213A), and hepatocellular carcinoma (Huh7). Flow cytometric research showed that the apoptosis pathway caused CCA cell death along with a reduction in procaspase-3, -8, and -9, an elevation in caspase enzymatic activity, and a decrease in anti-apoptosis Bcl-2 protein. Interestingly, phosphorylated-mTOR and phosphorylated-AKT proteins were significantly reduced after the extract treatment, indicating a strong reserve of AKT/mTOR survival signaling. Treatment with lutein and gallic acid significantly decreased the vitality of KKU100, KKU055, and KKU213A cells (Sawasdee et al., 2023). A myeloproliferative condition called chronic myeloid leukemia causes cells to create more ROS by activating many signaling pathways. Marine natural products have confirmed potential actions for the treatment of hematological malignancies, and nicotinamide adenine dinucleotide phosphate (NADPH) oxidases (NOXs) are a primary source of ROS in leukemia. Another research examined how the human leukemia cell line K562 was affected by the microalga *Skeletonema marinoi* Sarno and Zingone (Skeletonemataceae). After 48 h of treatment, S.M. was able to raise apoptosis levels and reduce cell viability in K562 cells. Furthermore, in K562-treated cells, SOD, CAT, and GPX levels rose while those of nitric oxide, NOX, and malondialdehyde decreased. Lastly, examining the gene expression of the proapoptotic protein Bax and the antiapoptotic protein Bcl-2, it was discovered that the K562-treated cells had a markedly higher level of Bax and a persistently lower level of Bcl-2 (Ciarcia et al., 2022).

Conventional medicine has been challenged by toxicity, drug resistance, and the inability of existing treatments to fully cure BC. As a result of its effectiveness and safety, supplementary alternative medicine has gained popularity. Carotenoids, which are known to suppress the development of cancer cells, are among the many bioactive found in the crude extract of *H. pluvialis* (H. pulvialis). In one research, the effects of H. pulvialis known as “T1″ on the BC cell line MDA-MB-231’s migration/invasion and cell proliferation were examined in the fibroblast control cells. TI caused apoptosis, prevented migration and invasion, and dramatically reduced the development of BC cells. Remarkably, an elevated Bax/Bcl2 ratio and a significant decrease of mutant p53 protein triggered apoptosis (Alateyah et al., 2022). Recent research indicates that because of its anti-inflammatory and antioxidant qualities, spirulina may have significant therapeutic effects. Another study’s main goal was to assess the chemopreventive properties of Spirulina microalgae (Spi) on regression and tumor survival, glioblastoma histological characteristics, and molecular mechanism detection. The MTT test was used to assess the tumor viability of Spi. Using the glioblastoma model, Spi’s *in vivo* anticancer efficacy was investigated. The animals were put to death and had their brains taken following tumor induction. The size and presentation of the tumor were assessed histologically. Through an assessment of the microRNAs and their targets, the processes behind Spi’s anticancer actions were examined. The findings showed that Spi caused cell death and suppressed the growth of C6 and U87 cells. According to histopathologic findings, giving Spi to animals with malignancies might postpone their growth and increase their survival time. Additionally, Spi markedly increased miR-125b and miR-34a, which are essential for the PI3K/AKT/mTOR pathway’s development (Arab et al., 2021).

Spirulina’s therapeutic anticarcinogenic efficacy against advanced mouse hepatocellular carcinoma was assessed in a study. Spirulina was administered orally in weeks 25 and 28 following the development of hepatocellular carcinoma at dosages of 250 and 500 mg/kg bw. By enhancing the survival rate, drastically lowering the tumor marker AFP, the number and size of hepatic nodules, and downstaging HCC, spirulina prevented structural and functional changes in HCC. Alongside this, apoptosis (Bax), tumor suppressor protein (p53), and endogenous antioxidant capability were all increased, while tissue levels of MDA and VEGF were decreased (Mahmoud et al., 2021). The purpose of another study was to examine the impacts of Schizochytrium sp. oil extract on the osteosarcoma cell line over time and in relation to dosage. Fatty acid samples at varying concentrations were applied to human fetal osteoblast cells and osteosarcoma cells. The fatty acid content of Schizochytrium sp. was analyzed using GC-FID. The findings showed that the oil extract sample inhibits cell growth and had a particular act against the SAOS-2 cancer line. The findings of the apoptosis experiment suggested that the oil extract sample had a selective effect on the bone cancer cell line (Sahin, 2021).

The microalgae of Mexico’s Cuatro Cienegas Basin are the subject of a bioprospecting investigation. Through rbcL gene sequencing and phylogenetic tree reconstruction, a microalga was identified as Granulocystopsis sp. Its anticancer properties were evaluated using a variety of *in vitro* assays and cell lines of human cancers, including breast, skin melanoma, lung, colorectal, and prostate cancers, in addition to a normal cell line. The clonogenic assay assessment revealed that the microalgae extract significantly inhibited cell proliferation and adhesion in cancer cell lines and produced a loss of membrane integrity *in vitro*. Also, characteristic nuclear alterations of apoptotic processes were seen under a microscope. Lastly, similar to the potent antitumoral medication doxorubicin, which is an apoptotic inductor, the microalgae extract elevated the action of caspases three and 7 in colon, breast, melanoma, and prostate cancer cells (Tavares-Carreón et al., 2020). The purpose of another study was to ascertain the antioxidant capacity of bioactive peptides obtained from 21-day-cultured *Synechococcus* sp. (Synechococcaceae) VDW cells. Synechococcus species. AILESYSAGKTK was the most effective antioxidant peptide for scavenging ABTS radicals out of the five that were found. Additionally, the untransformed Wi38 cell line was not affected by the FA fraction’s significant cytotoxic activity against human cancer-derived cell lines, particularly the colon cancer cell line (SW620). Following treatment for 24, 48, and 72 h, the FA fraction triggered the apoptotic pathway in SW620 cells; the greatest levels of caspases-3, -8, and -9 activity were seen following treatment for 72 h (Suttisuwan et al., 2019).

In a work, four distinct algal extracts, ethyl acetate, methanol, hexane, and chloroform, were tested for their ability to kill MCF-7, MDA-MB-231, A-549, and Hep-G2 cell lines. A variety of techniques, including the caspase-3 colorimetric test, AO/EB and Annexin V-FITC/PI double labeling, ROS, and MMP assay were used to validate the apoptosis. The greatest cytotoxic act against the cancer cell lines was demonstrated by ethyl acetate and methanol extracts, according to the data. The MDA-MB-231 cell line treated with ethyl acetate and methanol extracts showed a considerable increase in caspase-3 activity, ROS generation, and depolarized MMP (Abolhasani et al., 2018). Another study’s objective was to assess Nuevo Leon regional Chlorella sorokiniana’s *in vitro* cytotoxic impact. Ethidium bromide staining, acridine orange, and caspase activity were used to study the cell death process. The findings demonstrated that *C. sorokiniana* and *Scenedesmus* sp. methanol extracts significantly cytotoxically affected tumors through the mechanism of apoptosis (Reyna-Martinez et al., 2018).

The biological activities of the ethanolic extract of microalga were assessed in a different investigation. Bo tryidiopsidaceae sp. (ETBO) ethanol extract’s antioxidant activity was assessed using 1,1-diphenyl-2-picrylhydrazyl and free radical 2,2′-azino-bis (3-ethylbenzthiazoline-6-sulphonic acid) tests. The results showed that ETBO has concentration-dependent anticancer properties, including pro-apoptotic and anti-proliferation impacts on cancer cells, and antioxidant activity. Additionally, in response to ETBO, protein Bcl-2 expression reduced while p53 and caspase-3 expression rose dramatically. These findings suggested that ETBO activates caspase, which in turn triggers apoptosis, *via* modifying p53 and Bcl-2 (Suh et al., 2017a). A study made an ethanol extract of *Chloromonas* sp. (ETC.) and used human normal keratinocytes (HaCaT) and multiple

different cancer cell types, such as melanoma, cervical, and breast cancer cells (A375, HeLa, and Hs578T, respectively). It was shown that, ETCH has antioxidant properties and significantly inhibited the development of cancer cells and induced their death, but normal cells did not exhibit any anti-proliferation. Furthermore, ETCH significantly reduced cell invasion without having a deleterious impact. Additionally, in cancer cells treated with, ETCH, a reduction in Bcl-2 and an increase in cleaved caspase-3 and p53 caused ETCH-induced death (Suh et al., 2017b). In another work, the anticancer impacts of microalgae were evaluated utilizing a variety of assays and cell lines of human malignancies, like breast, prostate, lung, osteosarcoma, stomach, and pancreatic cancers. The microalgal extract demonstrated strong anti-colony formation action *in vitro*. Furthermore, it was more harmful to nonadherent cells than adherent cells, as seen by increased apoptosis. In NOD-SCID mice with subrenal capsule xenografts of PC3 prostate cancer cells, the extract showed an antimetastatic effect *in vivo*. The findings indicated that the aqueous microalgal extract’s antimetastatic action was predicated on the preferential destruction of suspended cancer cells and the reduction of cancer cells’ capacity to form colonies (Somasekharan et al., 2016).

Extract of Euglena tuba Ehrenberg (Euglenaceae) (ETME) was tested *in vitro* against human lung (A549) and breast cancer (MCF-7) cells to determine its antimetastatic and anticancer properties. Additionally, it has investigated how ETME regulates the MAPK pathway and antioxidants to produce intracellular ROS. The findings showed that ETME was non-toxic to normal WI-38 cells but hindered the development of both MCF-7 and A549 cells by causing apoptosis. BID truncation, caspase cascade activation, and an increase in the Bax/Bcl-2 ratio were the outcomes of ETME therapy. In both A549 and MCF-7 cells, this ultimately results in PARP breakdown and death *via* the intrinsic and extrinsic pathways. Downregulating ETME and MMP-9 dramatically reduced the invasion and migration of both A549 and MCF-7 cells in a dose-dependent manner. Subsequent research indicated that ETME inhibits the initiation of the JNK, ERK1/2, and P38 mitogen-activated protein kinase pathways in both selectively producing intracellular ROS, types of malignant cells, and controls intracellular antioxidant levels. Subsequent research on DNA and protein binding showed that ETME interacts strongly with both DNA and proteins, suggesting the presence of components that target the macromolecules in cancer cells (Panja et al., 2016). *Spirulina platensis* was tested for its ability to induce apoptosis in human lung cancer A-549 cell lines. A flash chromatography technology (Isolera system) was used to isolate the methyl ester of gamma linolenic acid, a significant omega-6 polyunsaturated fatty acid of therapeutic significance, from the microalgae *Spirulina platensis*. It was tested utilizing the SRB assay for *in-vitro* cytotoxic screening on A-549 lung cancer cell lines, and the outcome was contrasted with that of conventional rutin (Jubie et al., 2015).

The cytotoxicity of several crude solvent extracts from N. oculata and C. calcitrans against different cancer cell lines was examined in one research. After 72 h of treatment, the results showed that the crude ethyl acetate extract of C. calcitrans reduced the proliferation of MDA-MB-231 cells. The apoptosis determination test demonstrated that apoptosis was the primary mechanism of cell death. GeXP expression data revealed that B-cell leukemia/lymphoma 2 (Bcl-2) was downregulated while caspase-4 was increased (Goh et al., 2014). The ability of the stigmasterol that was separated from Navicula incerta to induce apoptosis in hepatocarcinoma cells was evaluated in a different investigation. The findings show that stigmasterol downregulated Bcl-2 and upregulated Bax and p53 most likely through the signaling mechanism for mitochondrial apoptosis. Nine caspases were activated when apoptosis was induced (Kim et al., 2014). Spirulina’s chemopreventive action against 7,12-dimethylbenz[a]anthracene (DMBA)-induced rat breast carcinogenesis was examined in a work, along with an investigation of its fundamental mechanisms of action *in vitro*. Spirulina curiously removed rat mammary tumors generated by DMBA, as demonstrated by morphological and histological techniques. The incidence of breast cancer decreased from 87% to 13% after taking supplements of spirulina. Immunohistochemical research showed that Spirulina supplementation decreased the expression of both estrogen alpha and Ki-67 at the molecular level. Also, SP administration decreased cell proliferation by 24 h. This was followed by an increase in p53 expression and higher expression of Cdkn1a (also known as p21 or p21(Waf1/Cip1)), the downstream target gene. Furthermore, Spirulina induced apoptosis 48 h after treatment by increasing Bax and decreasing Bcl-2 expression (Ouhtit et al., 2014). Another study evaluated the apoptotic mechanism and cytotoxic impact of crude ethanol extracts of Chaetoceros calcitrans on human breast cell lines. It was reported that EEC inhibited cell growth on MCF-7 cells by inducing apoptosis without cell cycle arrest. EEC modulated Cyclin A2, MDM2, CDK2, Bax, p21Cip1, and Bcl-2 to cause apoptosis in MCF-7 cells. Additionally, the EEC-treated MCF-7 cells had a higher Bax/Bcl-2 ratio, which triggered caspase 7 and the caspase-dependent pathways (Ebrahimi et al., 2013). Selected findings from *in vitro* and *in vivo* studies demonstrating the apoptotic effects of microalgae-derived compounds in various cancer models are summarized in Table 2. However, while these findings are promising, differences in study design, compound purity, and model systems highlight the need for standardized methodologies and mechanistic validation before clinical translation. Despite promising apoptotic effects, safety data are scarce. Certain formulations (e.g., nanoparticle conjugates) raise concerns about off-target toxicity and bioaccumulation. A balanced risk–benefit evaluation is therefore essential before therapeutic translation.

## 7 Future prospects and challenges of microalgae-derived compounds in cancer therapy

Despite growing interest in microalgae-based compounds, significant translational barriers remain that must be addressed to advance these findings toward clinical application. Microalgae-derived bioactive compounds have gained significant attention in recent years due to their potential to modulate apoptosis and inhibit tumor growth. Despite encouraging findings from numerous *in vitro* and *in vivo* studies, several critical challenges remain before these compounds can be effectively translated into clinical settings. One of the major challenges is the bioavailability and pharmacokinetics of these compounds. Many of them, particularly carotenoids like AST and FX, are poorly soluble in water and are rapidly metabolized in the body (Oninku et al., 2025). This limits their absorption and reduces their therapeutic efficacy. Current research is exploring ways to overcome these limitations, such as using lipid-based carriers or nanoformulations to improve stability and delivery. However, more work is needed to develop formulations that are both effective and suitable for human use. In addition to pharmacokinetic limitations, reported adverse effects and safety concerns must be considered. Some studies noted cytotoxicity to normal cells at higher doses, while others highlighted risks from nanoparticle-based delivery systems (Table 3).

**TABLE 3:** Reported adverse effects and safety considerations of microalgal metabolites.

Context	Reported issues	References
*In vitro* (normal cell lines)	Cytotoxicity at high concentrations (e.g., >100 μg/mL AST, FX extracts)	Suh et al. (2017b)
*In vivo* (animal models)	Some liver/kidney stress markers elevated at high doses; nanoparticle conjugates raised oxidative stress concerns	El-Baz et al. (2019)
Human supplementation trials	Generally well tolerated (e.g., *Phaeodactylum tricornutum* extract, *Chlorella pyrenoidosa* polysaccharides); mild GI discomfort occasionally reported	Qiu et al. (2022)
Environmental	Large-scale cultivation may affect ecosystems; contamination risks from heavy metals and microplastics	Kiki et al. (2022)

Another key issue is toxicity and safety, especially at therapeutic doses. While many studies highlight the selective toxicity of microalgae compounds toward cancer cells, some compounds, especially when delivered in crude extract or nanoparticle-conjugated forms, can still pose risks to healthy tissues. For example, silver nanoparticle formulations derived from Spirulina have shown potent anticancer activity, but their potential to induce oxidative stress or accumulate in organs remains a concern (Hussein et al., 2025). Systematic toxicity studies in animal models and eventually in human trials will be essential to clarify these risks. In addition, there are challenges related to standardization and reproducibility. The biochemical profile of microalgae can vary significantly depending on growth conditions, species, and extraction methods. This variability makes it difficult to ensure consistent therapeutic outcomes and poses a problem for large-scale production (Sandgruber et al., 2021). Integrating tools such as metabolomics and genomic analysis can help better characterize the active compounds and understand how to maintain their consistency across batches. Integrating omics technologies, such as proteomics, genomics, and metabolomics, could facilitate more precise identification and standardization of active compounds, improving reproducibility and therapeutic consistency (Saide et al., 2021). Large-scale cultivation of microalgae is another area that needs attention. While bioreactors and photobioreactor systems are promising, producing bioactive compounds in sufficient quantities for clinical use is still cost-intensive (Abdur et al., 2024). Efficient cultivation methods, harvesting techniques, and purification protocols must be optimized to make production scalable and economically feasible. Compared to bioactive compounds derived from terrestrial plants or fungi, microalgae offer distinct advantages including rapid growth rates, high biosynthetic efficiency, and adaptability to controlled environments, making them a promising and sustainable source of anticancer agents (Saide et al., 2021). This is especially important since many of the beneficial compounds are present in relatively low concentrations.

Despite these limitations, the future for microalgae-derived therapies looks bright. Advances in nanotechnology are already showing how targeted delivery systems can improve the precision and effectiveness of these compounds while minimizing side effects. Combining microalgae compounds with conventional treatments, such as chemotherapy or immunotherapy, has also shown promise in enhancing therapeutic outcomes and overcoming drug resistance. Compared to conventional chemotherapeutic agents, microalgae-derived compounds exhibit favorable selectivity toward cancer cells and are often associated with reduced systemic toxicity. This is largely attributed to their ability to modulate key apoptotic regulators such as Bax, Bcl-2, and caspases, without significantly affecting normal cells (Chiu et al., 2004). Additionally, several microalgae-derived bioactives have demonstrated potential in overcoming multidrug resistance by interacting with alternative signaling pathways distinct from those targeted by standard treatments. Despite these promising properties, most of these compounds remain in preclinical development, and data on their pharmacokinetic behavior and long-term safety are still limited (Bouyahya et al., 2024; Ahirwar et al., 2022). Therefore, while their potential as anticancer agents is evident, current evidence supports their use primarily as adjuvants to increase the efficacy and reduce the toxicity of established therapies, rather than as standalone alternatives (An et al., 2024). Finally, the lack of clinical trials is a major bottleneck. Although many preclinical studies report promising results, human trials are still scarce. Establishing the right dosages, understanding long-term effects, and evaluating interactions with other medications are all necessary steps before these compounds can be widely used in oncology. Addressing these safety, ecological, and standardization challenges is as critical as demonstrating efficacy. Only a balanced appraisal of both benefits and risks can guide rational development of microalgae-based therapies. It should be emphasized that these effects are not attributable to microalgae as a whole but to discrete bioactive molecules with defined structure–activity relationships. While Figure 3 provides an integrative framework, actual biological outcomes depend on the presence, concentration, and bioavailability of specific compounds. Broad generalizations about “microalgae” should therefore be avoided, and further mechanistic validation is required to delineate structure–activity relationships that underpin their pharmacological actions.

In summary, while microalgae hold great promise as a source of novel anticancer agents, their successful integration into clinical practice will depend on overcoming several scientific and logistical barriers. Addressing issues like bioavailability, toxicity, reproducibility, and scalability, along with a stronger focus on translational research, will be key to unlocking their full therapeutic potential. Overall, the translational value of current data is constrained by small sample sizes, limited human studies, and incomplete safety assessments, underscoring the need for standardized preclinical protocols and rigorous clinical trials.

## 8 Limitations and future research

This review, and the wider field, is limited by the heterogeneity and generally modest quality of available studies. Most mechanistic insights are derived from *in vitro* cancer cell or animal models, with limited translation to human trials. Many reports rely on single-cell line assays without replication, selectivity indices, or standardized controls, raising concerns about reproducibility and clinical relevance. Furthermore, bioavailability challenges remain a significant barrier to clinical application, and few studies have investigated long-term safety or optimal dosing in humans. The pharmacological effects discussed also arise from individual metabolites rather than from microalgae universally, underscoring the need for careful structure–activity studies to strengthen mechanistic understanding and support translational relevance. In addition, adverse effects and ecological implications of large-scale microalgae cultivation remain poorly studied and must be integrated into future risk–benefit assessments. Future research should therefore prioritize standardized experimental protocols, mechanistic validation across multiple models, and rigorously designed human trials that report not only efficacy but also safety, tolerability, and long-term outcomes.

## 9 Conclusion

Microalgae-derived metabolites are an emerging but still underexplored group of natural compounds with potential importance for both neurodegenerative diseases and cancer. Key examples include fucoxanthin, astaxanthin, phycocyanin, and omega-3 fatty acids, which have been shown to exert antioxidant, anti-inflammatory, pro-apoptotic, and neurogenic effects. Their ability to influence both brain and tumor metabolism highlights their promise as dual modulators of apoptosis and neuroplasticity. Although interest in these compounds is growing, most studies focus on either neurodegeneration or cancer in isolation. This review takes one of the first steps toward integrating these two areas, emphasizing shared molecular pathways—such as mitochondrial dysfunction, oxidative stress, and disrupted cell signaling—that can be targeted by microalgal metabolites. Exploring these intersections may open new therapeutic opportunities.

From a practical perspective, microalgae offer a sustainable and versatile source of multifunctional bioactive compounds that could complement existing therapies. Advances in nanotechnology and delivery systems may further improve the stability, bioavailability, and precision of these agents, helping to bring them closer to clinical use. Overall, targeting brain and tumor metabolism through microalgal metabolites represents a compelling strategy to address both cancer progression and neurodegenerative disorders. Their broad biological activity, favorable safety profile, and natural origin make them attractive candidates for inclusion in personalized treatment approaches. Looking ahead, well-designed mechanistic studies and human clinical trials will be crucial to confirm efficacy, establish safety and tolerability, and evaluate ecological sustainability. Only then can microalgal bioactives be realistically positioned as adjuncts or novel agents in oncology and neurology.
